# Citalopram amplifies the influence of living conditions on mood in depressed patients enrolled in the STAR*D study

**DOI:** 10.1038/tp.2017.35

**Published:** 2017-03-21

**Authors:** F Chiarotti, A Viglione, A Giuliani, I Branchi

**Affiliations:** 1 Center for Behavioral Sciences and Mental Health, Istituto Superiore di Sanità, Rome, Italy; 2 Department of Environment and Health, Istituto Superiore di Sanità, Rome, Italy; 3 Institute of Anatomy, University of Zurich, Zurich, Switzerland

## Abstract

Selective serotonin reuptake inhibitors (SSRIs), the most commonly prescribed antidepressant drugs, have a variable and incomplete efficacy. In order to better understand SSRI action, we explored the hypothesis that SSRIs do not affect mood *per se* but amplify the influence of the living conditions on mood. To this aim, we exploited the Sequenced Treatment Alternatives to Relieve Depression (STAR*D) data set, selected a subpopulation of 591 patients with an overlapping clinical history and analyzed treatment outcome according to dosage −20 or 40 mg per day of citalopram. We found that sociodemographic characteristics affected treatment response in the same direction in the two dose groups, but these effects reached statistical significance only in the 40 mg per day dose group. In the latter, higher improvement rate was associated with having a working employment status (*P*=0.0219), longer education (*P*=0.0053), high income (*P*=0.01) or a private insurance (*P*=0.0031), and the higher remission rate was associated with having a working employment status (*P*=0.0326) or longer education (*P*=0.0484). Moreover, the magnitude of the effect of the sociodemographic characteristics on mood, measured as the percent of patients showing a positive outcome when exposed to favorable living conditions, was much greater—up to 37-fold—in the 40 compared to the 20 mg per day dose group. Overall, our results indicate that citalopram amplifies the influence of the living conditions on mood in a dose-dependent manner. These findings provide a potential explanation for the variable efficacy of SSRIs and might lead to the development of personalized strategies aimed at enhancing their efficacy.

## Introduction

Antidepressant drugs are the current standard treatment for major depressive disorder (MDD) and, among these, selective reuptake inhibitors (SSRIs) are the most commonly prescribed. However, their efficacy is variable and incomplete: 60–70% of depressed patients do not experience remission and 30–40% do not show a significant response.^1^ One of the main reasons for such incomplete efficacy is the poor comprehension of their mechanisms of action. A new hypothesis, the undirected susceptibility to change hypothesis, predicts that SSRI treatment does not drive changes in mood *per se* but, by increasing brain plasticity, creates a window of opportunity for a change in mood that is driven by the quality of the living conditions.^2^ In particular, the serotonin increase induced by SSRIs enhances brain plasticity and thus renders the individual more susceptible to the influence of the living conditions. The main consequence is the lack of a univocal outcome of SSRI administration: in a favorable environment treatment leads to a reduction of symptoms; by contrast, in a stressful environment it may lead to a worse prognosis. As a further consequence, SSRIs are expected to amplify the influence of living conditions on mood in a dose-dependent manner. Such hypothesis is supported by preclinical data^3, 4, 5^ showing that fluoxetine treatment leads to an improvement of the depression-like phenotype when administered in an enriched condition, while it leads to a worsening when administered in a stressful condition. In addition, SSRI treatment consequences on selected end points, such as vulnerability to obesity, have been shown to be dependent on the quality of the environment.^6, 7^ Finally, a number of clinical studies have shown that the environment moderates the effects of antidepressant treatment.^1, 8, 9, 10, 11, 12, 13^ However, the approaches used so far in these studies do not allow for assessing the effect of SSRIs on the susceptibility to the influence of the living conditions.

The main aim of the present study was to test whether SSRI treatment amplifies the influence of the living conditions on mood in a dose-dependent manner. We thus exploited the data collected in the framework of the Sequenced Treatment Alternatives to Relieve Depression (STAR*D) study. We considered a subpopulation of 591 patients having similar MDD severity and overlapping treatment history, and analyzed the efficacy of treatment between weeks 4 and 6 according to the dose received—either 20 or 40 mg per day of citalopram. Longer or different treatment periods or other patients' groups could not be considered without losing data validity because of the limitations imposed by the STAR*D trial design. The socioeconomic characteristics included in the analysis are proxy of the quality of the patient's life environment and widely considered reliable indicators of socioeconomic status.^14^ Our prediction was that the 40 mg per day dose, compared to the 20 mg per day dose, would amplify the influence of sociodemographic characteristics on patients' mood, since it should increase plasticity to a higher degree and thus lead to greater susceptibility to the environment. We therefore predicted citalopram to affect susceptibility to the living conditions in a dose-dependent manner. In addition, since we hypothesized that the environment drives the change in MDD induced by SSRIs, we predicted that patients living in conditions associated to a high quality of life should show a more effective response to treatment.

## Materials and methods

### Study organization

This paper is based on the STAR*D (ClinicalTrials.gov, number NCT00021528) study data. The design details of STAR*D are described accurately elsewhere.^15^ In brief, STAR*D was a multisite, prospective, randomized, multistep clinical trial conducted in the United States of America aimed to determine which of several treatments would be most effective for outpatients with nonpsychotic MDD.^16^ The study was conducted at 18 primary care and 23 psychiatric care centers and enrolled 4041 nonpsychotic MDD patients, aged 18–75, with a baseline score ⩾14, on the 17-item Hamilton Depression Rating Scale (HAM-D17).

MDD symptom severity in the STAR*D clinical trial was measured longitudinally using the 16-item Quick Inventory of Depressive Symptomatology (QIDS). The QIDS is a briefer version of the more commonly used 30-item Inventory of Depressive Symptomatology—IDS. The QIDS is available in the clinician and self-rated version and has been designed to assess the severity of depressive symptoms through the evaluation of all the criterion symptom domains designated by the American Psychiatry Association Diagnostic and Statistical Manual of Mental Disorders—4th edition (DSM-IV) to diagnose a major depressive episode. Each item was scored on a scale from 0 to 3 points: 0 indicating no problem and 3 indicating severe problem. Total score ranges from 0, that is, not depressed, to 27, that is, most depressed. For further details on the validity, reliability, generalizability, scoring and interpretation, see http://www.ids-qids.org/.

### Treatment

At Level 1, all participants were treated with citalopram, a SSRI, for a minimum of 8 weeks and were encouraged to complete 12 weeks to maximize benefit. All participants started treatment with a dose of 20 mg per day citalopram, with clinical visits at 2, 4, 6, 9 and 12 weeks. To ensure satisfactory dosing for an appropriate period of time, treatment was conducted using measurement-based care. This included flexible dosing recommendations based on symptom and side effect at each treatment visit.^17^ According to the *Guidelines for discontinuing participants from the randomized treatment study*, patient drop out could be due to a number of reasons including participant request and decision by clinicians to discontinue the study is in the best interest of the participant.

Dose adjustments were decided according to the QIDS-Clinician-rate (QIDS-C16) score: QIDS-C16⩽5, continue current dose; QIDS-C16=6–8, continue or increase current dose according to the clinician assessment; QIDS-C16⩾9, increase current dose. In addition, if the reduction in baseline symptom severity was found to be <20% at week 4, the initial dose (20 mg per day) was raised to 40 mg per day, assuming tolerable side effects. At week 4, participants with intolerable side effects could move to the next treatment level.^17^

### Selection criteria

In the present study, data from level 1 of STAR*D were considered. Only patients having similar MDD severity and overlapping treatment history were included in the analysis. In particular, we selected patients treated with a dose of 20 mg per day for the first 4 weeks following enrollment, showing a QIDS-SR16 score equal to 6–10 (mild depression) on week 4 and whose information concerning the QIDS score on week 6 was available (Supplementary Figure S1). Longer or different treatment periods or other patients' groups could not be considered without losing data validity because of the limitations imposed by the STAR*D trial design. In particular, according to the recommendations of the clinical trial design, patients with high QIDS score that indicates moderate, severe and very severe depression are compulsorily assigned to a dose increase. Including patients with all QIDS scores in the analysis would lead to bring severe depressed and potentially non-responding patients in the high dose group, erroneously leading to the conclusion that the high dose is less effective. The subset of patients considered in the present study show no meaningful clinical difference in depressive symptomatology (measured as QIDS-SR16 score at enrollment, at week 4 and as difference between week 4 and enrollment) according to the dose assignment (see Results section and Table 1). We therefore analyzed treatment efficacy between weeks 4 and 6 according to the dose received, either 20 or 40 mg per day of citalopram.

### Sociodemographic characteristics

The efficacy of the two dosing regimens was analyzed in relation to a number of sociodemographic characteristics considered as proxy of the socioeconomic status and the quality of the living environment of the patient:^14^ sex, race, marital status, employment status, insurance status, education, income, experience of traumatic events and drug abuse. In addition, we analyzed the following descriptors of the onset and progression of the psychopathology: age at onset of first major depressive episode (MDE), number of MDE, difference in QIDS-SR16 score between enrollment and week 4. *Education* was shown as years of schooling completed; we considered two levels of education: <college (<16 years) and ⩾college (⩾16 years). *Income* was analyzed subdividing the examined population into three monthly income classes: the low-income group was set at ⩽$1000, while middle and high income groups at $1000–$2500 and >$2500, respectively. *Employment* status was analyzed considering only two conditions: employed (self-employed, part-time employed and full-time employed) and unemployed (unemployed and retired). For marital status we considered: never married, married (married or cohabiting) and no more married (separated, divorced and widowed). Finally, we considered only two ethnicities, Caucasian (white) and non-Caucasian (all other ethnicities).

### Outcome measures

MDD symptom severity was measured using the QIDS-SR16. Remission was defined as a QIDS-SR16 score ⩽5. In order to determine the effects of citalopram treatment, according to sociodemographic characteristics, we considered three variables: (i) percent of patients showing an improvement, measured as a reduction ⩾1 in QIDS-SR16 score between week 4 and 6; (ii) percent of patients achieving remission, measured as the attainment of a QIDS-SR16 score ⩽5 at week 6; and (iii) variation in the QIDS-SR16 score, measured between weeks 4 and 6.

### Data analysis

Of the 4041 participants, a sample of 591 patients was identified according to the selection criteria (Supplementary Figure S1). Summary statistics are presented as percentages for discrete variables, and as means and s.d. for continuous variables. Logistic regression models were used to assess the association of all sociodemographic characteristics with QIDS-SR16 score improvement and remission rate, within each dose group, and to compute odds ratios. The Wald test was used to compute the overall significance of the categorical sociodemographic factors presenting more than two levels. The use of the odds ratios as a measure of risk is debatable and its approximation to the relative risk (RR) is acceptable only when the risk of the event (in the present study, the improvement or remission) in the control group (in the present study, the favorable sociodemographic condition) is very low, that is, under the so-called 'rare disease assumption'.^18^ For this reason, we additionally computed the percent of improvement or remission in the favorable (that is, associated to a high quality of life) and in the unfavorable (that is, associated to a low quality of life) sociodemographic condition, and the RR of unfavorable versus favorable condition, with the corresponding 95% confidence interval. Finally, the preventive fraction (1−RR) was computed to estimate the effect size and thus the magnitude of influence of the living conditions on the outcome. As in our study the outcome measured is a beneficial one, that is improvement or remission, values of 1−RR>0 indicate that the unfavorable condition decreases the probability of the beneficial outcome compared to the favorable condition, and *vice versa.* The variation in the QIDS-SR16 score between weeks 4 and 6 was analyzed with analyses of variance including sociodemographic characteristics as between-subject factors. Separate analyses of variance were performed for each sociodemographic characteristic within the two treatment doses. *Post hoc* comparisons were performed using the Tukey's test. The variation in percent of improvement, stationary and worsening according to sociodemographic characteristics has been analyzed independently in the two dose groups with *X*^2^-test.

## Results

### Baseline characteristics

Of 591 patients who comprised the evaluable sample, 357 (60.4%) were treated with 20 mg of citalopram per day and 234 (39.6%) with 40 mg of citalopram per day between weeks 4 and 6. Females comprised two-thirds of the sample and minority representation was robust. The two dose groups did not show any meaningful clinical difference before receiving different citalopram doses (Supplementary Table S1). At enrollment, their QIDS-SR16 scores were almost overlapping (20 mg per day dose group: mean 14.14, s.d. 3.78; 40 mg per day dose group: mean 14.7, s.d. 3.79). The two groups did not differ also in QIDS-SR16 score at week 4 (20 mg per day dose group: mean 7.93, s.d. 1.39; 40 mg per day dose group: mean 8.16, s.d. 1.44). Finally, as shown in Table 1, no difference between the 20 and 40 mg per day dose groups was found in response to treatment as both showed an almost overlapping reduction of QIDS-SR score during the first 4 weeks of treatment, when all patients received the 20 mg per day dose. Table 1 summarizes further baseline characteristics of the two groups.

### Sociodemographic characteristics and dosage associated with improvement

According to our hypothesis, the two citalopram dosages amplified the influence of sociodemographic characteristics on the percent of patients showing an improvement in a dose-dependent manner. In the 20 mg per day dose group, patients' response was not significantly affected by sociodemographic characteristics. By contrast, in the 40 mg per day dose group, sociodemographic characteristics were associated to significantly different outcomes. In the latter group, a higher rate of improvement was associated with having a working employment status, more than 16 years of education, high income and a private insurance (Table 2). In order to appreciate the influence of the environment in each dose group, its magnitude was measured as the preventive fraction (1−RR). The comparison of the preventive fraction, indicating the percent of patients showing a positive outcome when exposed to a favorable environment, between the two dose groups shows that the influence of sociodemographic characteristics on treatment outcome was overall markedly higher in the 40 mg per day dose group. In particular, the magnitude of the influence of the employment status (unemployed versus employed) and education (<college versus ⩾college) on improvement rate was, respectively, 5- and 37-fold in the 40 compared to the 20 mg per day dose group. In addition, the magnitude of the influence on improvement rate of income was 6- (high versus low) and 8-fold (high versus middle) and of insurance was 22- (no versus public) and 8-fold (private versus public) in the 40 compared to the 20 mg per day group (Supplementary Table S2).

### Rate of remission

As for the rate of improvement, the two citalopram dosages produced a different amplification of the influence of the sociodemographic characteristics on the percent of patients achieving remission. In particular, in the 40 mg per day group, a significantly higher rate of remission was found to be associated with being employed and having longer education (Table 3). The magnitude of the influence of the employment status (unemployed versus employed) and education (<college versus ⩾college) on remission rate was, respectively, six- and twofold in the 40 compared to the 20 mg per day group (Supplementary Table S3).

### Variation in the QIDS-SR16 score

**Figure 1:**
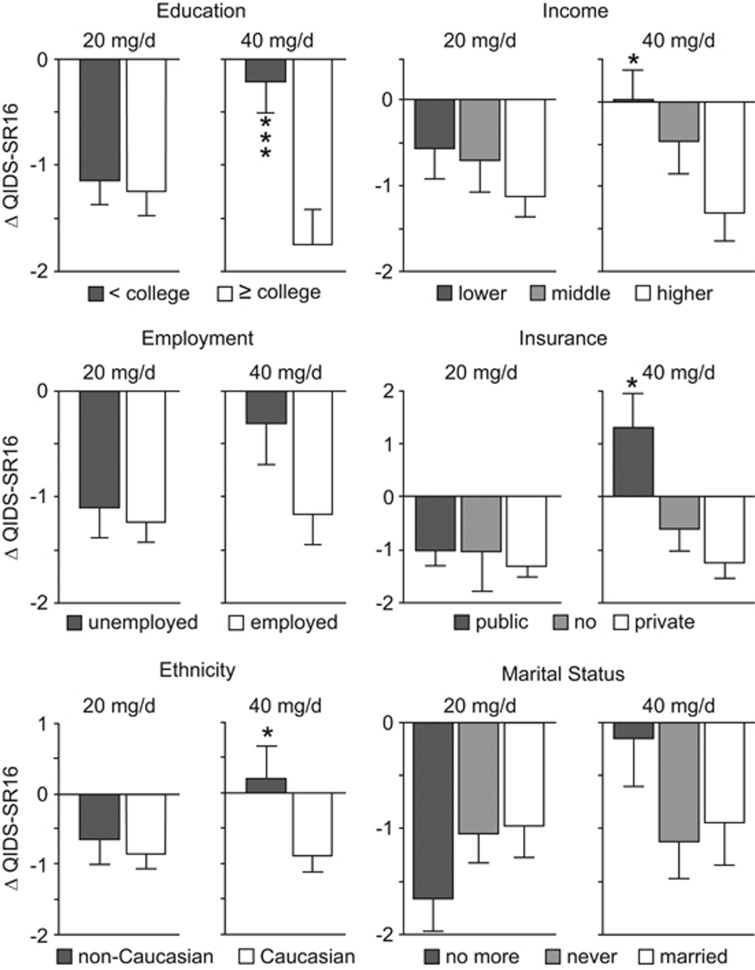
Variation in the QIDS-SR16 score, measured between weeks 4 and 6, in patients receiving either the 20 or 40 mg per day dose. Negative values indicate a reduction of the QIDS-SR16 score, that is, an improvement of the patient's mental health condition. Only patients receiving the 40 mg per day dose showed a variation in the QIDS-SR16 significantly moderated by the sociodemographic characteristics. *Indicates difference with the first group; *P<0.05 and ***P<0.001. QIDS-SR16, Quick Inventory of Depressive Symptomatology Self-Report.

The variation in the QIDS-SR16 score produced results in line with the previous ones, as it significantly differed according to sociodemographic variables only in the 40 mg per day dose group. In particular, in this group, being of Caucasian ethnicity (F (1,232)=5.334, *P*=0.0218), having a private insurance (F (2,185)=4.427, *P*=0.0132), a high income (F (2,225)=3.629, *P*=0.0281) or more years of education (F (1,187)=11.344, *P*=0.0009) was associated with a significant larger reduction of QIDS-SR16 score (Figure 1). *Post hoc* analysis revealed a significant reduction of the QIDS-SR16 comparing high versus low income (*P*<0.05) and having a private versus a public insurance (*P*<0.05).

## Discussion

The results of the present study show that citalopram amplifies the influence of the living conditions on mood in a dose-dependent manner as sociodemographic characteristics modify treatment response in the same direction in the two dose groups, but in the 40 mg per day dose group the effect is much larger and reaches statistical significance. In addition, the magnitude of the influence of the living conditions on mood is much greater—up to 37-fold—in the 40 compared to the 20 mg per day dose group. These results support the undirected susceptibility to change hypothesis,^2^ which predicts that, as SSRI increases the susceptibility to the environment, treatment outcome is more profoundly affected by the quality of the living conditions in patients receiving high dosages of SSRIs.

The STAR*D clinical trial provides a unique opportunity to investigate the role of citalopram as amplifier of the influence of the living conditions on mood. It has allowed to consider a subpopulation of patients with similar MDD severity and overlapping treatment history in order to analyze the amplification of the influence of the sociodemographic features induced by different dosages of citalopram. Given the clear ethical limitations to perform a clinical trial aimed at directly measuring the effects of the SSRIs in amplifying the beneficial, but especially the detrimental effects of the environment in patients, the STAR*D data set is the best alternative to test the undirected susceptibility to change hypothesis. In addition, the STAR*D clinical trial is the largest ecologically valid 'real world' study of outpatients with nonpsychotic major depressive disorder to date.^1, 19^

Sociodemographic characteristics modified treatment outcome in the same direction in the two dose groups, but these changes did not reach statistical significance in the 20 mg per day dose group. By contrast, in the 40 mg per day dose group, each one of five sociodemographic characteristics—income, education, ethnicity, insurance and employment—significantly affected treatment outcome. In addition, the magnitude of the effect of the sociodemographic characteristics on mood, measured as the percent of patients showing a positive outcome when exposed to a favorable environment, was dose-dependent. In particular, the influence of the living conditions was much greater in the 40 than in the 20 mg per day dose group: improvement rate went from a minimum of fivefold for employment status to a maximum of 37-fold for education while remission rate went from twofold for education to eightfold for income.

It is worth noting that, in line with our hypothesis and with previous studies showing that a favorable and supportive environment increases antidepressant efficacy,^1, 10, 11, 13, 20^ the present results indicate not only that citalopram amplifies the influence of the living conditions on mood, but also that the quality of the living environment drives the change in mood. In particular, in the 40 mg per day dose group, where this change reaches statistical significance, improvement and remission were shown at significantly higher rates by patients living in conditions associated with a high quality of life, such as having a working employment status, more than 16 years of education and a high income. The exception concerns insurance where those individuals having public insurance showed the worse outcome, even compared to those having no insurance.^21^ This is concordant with previous studies reporting, for instance, that having public insurance predicts the highest attrition.^17^ In addition, higher rates of improvement and remission were associated with being of Caucasian ethnicity. The sociodemographic characteristics here found to affect treatment outcome have been previously shown to both determine rates of major depression morbidity and mortality^22^ and affect SSRI outcome.^1^ According to our hypothesis,^2^ even a worsening of symptomatology could be predicted when citalopram treatment is administered in an adverse environment. However, only a very limited worsening of the QIDS-SR score was expected in patients receiving the treatment while living in an unfavorable condition (Figure 1) because of the implementation of the STAR*D guidelines for discontinuing participants, which recommend that patients drop out of the study when clinicians consider that discontinuation is in the best interest of the participant. Nevertheless, an effect of treatment, both for better and for worse,^23^ can be appreciated when considering the percent of patients showing improvement or worsening according to the quality of the living conditions in the two dose groups (Supplementary Figure S2). Indeed, the 40, compared to 20 mg per day dose, leads to an increase not only of the percent of improvements in patients living in a favorable environment, but also of the percent of worsening in patients living in an unfavorable environment. For instance, in the 20 mg per day dose group, patients with a college degree showing an improvement were 62% and patients with a high school degree showing a worsening were 21%. In the 40 mg per day group, these percent rise up, respectively, to 71% and 35%. As further example, in the 20 mg per day dose group, patients with a high income showing an improvement were 58% and patients with a low income showing a worsening were 35% while, in the 40 mg per day group, these are, respectively, 68% and 40% (Supplementary Figure S2).

Taking into account the role of SSRIs as amplifier of the influence of living conditions on mood—and, consequently, the quality of the environment as a moderator of SSRI treatment outcome^24^—may explain the inconsistency of the findings in the literature about SSRI efficacy. Indeed, overlooking the environment as the key stratifying factor in treatment response might have led to the reported contradictory results in antidepressant outcome. The conceptual shift in considering the action of SSRIs from being the causative factor for recovering from major depression to acting as a permissive factor for the influence of the living conditions on the psychopathology allows to reconcile experimental and clinical data that apparently do not fit together.^2^ For instance, the theoretical framework currently available in the literature leads to the paradox that the same molecular mechanism of action has two opposite outcomes: high extracellular serotonin levels are beneficial when induced by SSRI administration, but confer a high risk to develop psychopathology when associated to the *s* variant of the serotonin-transporter-linked polymorphic region (5-HTTLPR). This discordant picture can be coherently interpreted in light of our results and the undirected susceptibility to change hypothesis, positing that high serotonin levels lead to increased plasticity and thus to high susceptibility to change, which may promote either an improvement or a worsening, according to the quality of the environment.^2^ It is worth noting that the effects of citalopram described in the present paper may represent only part of the action of SSRIs on mood as the main target of these drugs, that is, the serotoninergic system, has a high molecular complexity and is involved in a wide range of physiological functions.

The major limitation of the present study is the short time frame (2-week period) over which data of the STAR*D clinical trial have been extracted. This period had to be chosen because it is the only one allowing to consider patients with overlapping clinical history. Longer or different treatment periods or other patient groups would have not allowed to keep data validity. By contrast, the fact that coherent significant results have been found in such a short time frame suggests that the described phenomenon is robust. Further limitations include open treatment design, the use of a single antidepressant agent (citalopram) and the lack of placebo control. Although data analyses did not include correction for multiple comparisons, the overall consistence of the results support their reliability. It is worth noting that depression treatment disparities experienced by different ethnicities may result from stigma, clinician failure to engage with the patient, poor patient activation, treatment adherence and other factors, including biological differences.^25^

In conclusion, acknowledging the role of SSRIs as amplifier of the influence of the living conditions on mood represents a critical step in developing a personalized medicine approach aimed at better matching patients with treatment and avoiding potential harmful consequences. The control of the patients' living environment could be achieved by training patients to cope with harsh conditions, for instance, through cognitive behavioral therapy,^26^ or taking them in charge through appropriate specialized services, as it is unlikely that people can rapidly and effectively change their living milieu. The cost of this approach is limited as no new psychoactive molecules need to be developed, while the benefits for the patients could be substantial. Finally, the undirected susceptibility to change hypothesis may contribute building a new theoretical framework capable to integrate the 'chemical imbalance theory' with other hypotheses acknowledging the importance of social–psychological factors in MDD, as both approaches are needed to explain the mechanisms underlying the recovery from the disease.

## Figures and Tables

**Table 1:** Baseline sociodemographic characteristic of the evaluable sample by citalopram dose

*Baseline characteristics*		*Citalopram dose*	
	N=*591 (%)*	*20 mg per die;* N=*357 (60.4%)*	*40 mg per die;* N=*234 (39.6%)*
*Ethnicity*			
Non-Caucasian^a^	165 (27.9)	108 (30.3)	57 (24.4)
Caucasian	426 (72.1)	249 (69.7)	177 (75.6)
			
*Sex*			
Female	362 (61.3)	222 (62.2)	140 (59.8)
Male	229 (38.7)	135 (37.8)	94 (40.2)
			
*Marital status*			
No more married	125 (25.8)	77 (26.1)	48 (25.4)
Never married	140 (28.9)	86 (29.2)	54 (28.6)
Married	219 (45.2)	132 (44.7)	87 (46.0)
			
*Employment status*			
Unemployed and retired	185 (38.2)	110 (37.3)	75 (39.7)
Employed	299 (61.8)	185 (62.7)	114 (60.3)
			
*Education*^b^			
<College	294 (60.7)	180 (61.0)	114 (60.3)
⩾College	190 (39.3)	115 (39.0)	75 (39.7)
			
*Income*^c^			
Low	172 (29.9)	105 (30.3)	67 (29.4)
Middle	195 (33.9)	114 (32.9)	81 (35.5)
High	208 (36.2)	128 (36.9)	80 (35.1)
			
*Experienced traumatic event*			
No	333 (56.8)	203 (57.3)	130 (56.0)
Yes	253 (43.2)	151 (42.7)	102 (44.0)
			
*Witnessed traumatic event*			
No	405 (69.1)	241 (68.1)	164 (70.7)
Yes	181 (30.9)	113 (31.9)	68 (29.3)
			
*Drug abuse*			
No	129 (95.6)	73 (97.3)	56 (93.3)
Yes	6 (4.4)	2 (2.7)	4 (6.7)
			
*Insurance status*			
Public insurance	39 (8.0)	25 (8.6)	14 (7.4)
No insurance	179 (36.8)	103 (35.3)	76 (40.4)
Private insurance	269 (55.2)	164 (56.2)	98 (52.1)

	*Median (observed range)*	*Mean (s.d.)*	*Mean (s.d.)*	*Mean (s.d.)*
Age (years)	41.0 (18–75)	41.3 (13.3)	41.3 (13.6)	41.1 (12.8)
Years of education	14.0 (1–25)	14.3 (9.5)	14.2 (3.4)	14.5 (3.0)
Income ($ per month)	1 900 (0–50 000)	2794 (3620)	2670 (2785)	2982 (4610)
Age at onset of first MDE	22.0 (4–73)	26.3 (14.6)	27.0 (15.1)	25.1 (13.8)
Number of MDEs	3.0 (1–84)	5.6 (9.1)	5.3 (7.7)	6.0 (10.8)
QIDS-SR_16_ (difference W4−Enroll)	−6 (−19–7)	−6.4 (3.9)	−6.2 (3.9)	−6.6 (3.9)

Abbreviations: MDE, major depressive episode; QIDS-SR16, Quick Inventory of Depressive Symptomatology-Self-Report.

a Asian, American Indian or Alaskan Native, Native Hawaiian/Other Pacific Islander or multiracial.

b <College=⩽16 years of schooling; ⩾College=⩾16 years of schooling.

c Low=monthly gain ⩽$1000; Middle=monthly gain $1000–$2500; High=monthly gain >$2500.

**Table 2:** Baseline sociodemographic characteristics associated with percentage of patients improved by citalopram dose^a^

*Characteristics*	*20 mg per die;* N=*357 (60.4%)*		*OR*^b^	P*-value*	*40 mg per die;* N=*234 (39.6%)*		*OR*^b^	P*-value*
	*Improved %*	*Not improved %*			*Improved %*	*Not improved %*		
Overall	56.9	43.1			54.7	45.3		
								
*Ethnicity*				0.5750				0.1150
Non-Caucasian^c^	54.6	45.4	0.88		45.6	54.4	0.62	
Caucasian	57.8	42.2			57.6	42.4		
								
*Sex*				0.4069				0.2371
Female	58.6	41.4	1.20		57.9	42.1	1.37	
Male	54.1	45.9			50.0	50.0		
								
*Marital status*				0.1300				0.8100
No more married	71.4	28.6	1.68		54.2	45.8	0.80	
Never married	57.0	43.0	0.89		59.3	40.7	0.98	
Married	59.9	40.2			59.8	40.2		
								
*Employment status*				0.5790				0.0219
Unemployed and retired	60.0	40.0	0.87		48.0	52.0	0.50	
Employed	63.2	36.8			64.9	35.1		
								
*Education*^d^				0.9336				0.0053
<College	62.2	37.8	1.02		50.0	50.0	0.42	
⩾College	61.7	38.3			70.7	29.3		
								
*Income*^e^				0.7200				0.0100
Low	54.3	45.7	0.87		43.3	56.7	0.37	
Middle	59.7	40.4	1.08		50.6	49.4	0.49	
High	57.8	42.2			67.5	32.5		
								
*Experienced traumatic event*				0.4731				0.2100
No	58.1	41.9	1.17		51.5	48.5	0.71	
Yes	54.3	45.7			59.8	40.2		
								
*Witnessed traumatic event*				0.2660				0.1130
No	58.5	41.5	1.29		51.8	48.2	0.63	
Yes	52.2	47.8			63.2	36.8		
								
*Drug abuse*				0.9540				0.2950
No	52.1	48.0	1.09		46.4	53.6	0.29	
Yes	50.0	50.0			75.0	25.0		
								
*Insurance status*				0.8300				0.0031
Public insurance	68.0	32.0	1.33		14.3	85.7	0.08	
No insurance	62.1	37.9	1.02		53.9	46.1	0.57	
Private insurance	61.6	38.4			67.3	32.7		

	*Mean (s.d.)*	*Mean (s.d.)*	*OR*^b^	*P-value*	*Mean (s.d.)*	*Mean (s.d.)*	*OR*^b^	P*-value*
Age (years; units=10)^f^	40.6 (13.5)	42.3 (13.7)	0.91	0.2326	40.2 (12.6)	42.2 (12.9)	0.88	0.2200
Years of education (units=4)^f^	14.2 (3.4)	14.3 (3.3)	0.98	0.8610	14.9 (3.0)	13.9 (2.9)	1.53	0.0402
Income ($ per month; units=1000)^f^	2718 (2644)	2606 (2973)	1.01	0.7100	3483 (5446)	2385 (3279)	1.08	0.0940
Age at onset of first MDE (units=10) f	26.8 (14.6)	27.3 (15.8)	0.98	0.7860	24.7 (13.2)	25.5 (14.6)	0.96	0.6630
Number of MDEs (units=5) f	4.9 (8.3)	5.7 (6.9)	0.92	0.2287	4.6 (6.3)	7.7 (14.6)	0.86	0.0436
QIDS-SR_16_ (difference W4−Enroll; units=1)^f^	−6.1 (4.1)	−6.3 (3.7)	1.01	0.7080	−5.7 (3.8)	−7.6 (3.8)	1.13	0.0005

Abbreviations: MDE, major depressive episode; OR, odds ratio; QIDS-SR16, Quick Inventory of Depressive Symptomatology-Self-Report.

a Improvement was measured as a reduction ⩾1 in 16-item QIDS-SR_16_ score between weeks 4 and 6.

b For characteristics for which the measurement is categorical, the comparison is with the group listed last under the category or with the opposite characteristic. For characteristics for which the measurement is continuous, the odds ratio is relative to an increase in the measurement by the number of units indicated.

c Asian, American Indian or Alaskan Native, Native Hawaiian/Other Pacific Islander or multiracial.

d <College=⩽16 years of schooling; ⩾College=⩾16 years of schooling.

e Low=monthly gain ⩽$1000, Middle=monthly gain $1000–$2500, High=monthly gain >$2500.

f Units are relevant to odds ratios only OR, MDE.

**Table 3:** Baseline sociodemographic characteristics associated with remission by citalopram dose^a^

Characteristics	*20 mg per die;* N=*357 (60.4%)*		*OR*^b^	*P-value*	*40 mg per die*; N=*234 (39.6%)*		*OR*^b^	*P-value*
	*REM %*	*NO REM %*			*REM %*	*NO REM %*		
Overall	35.9	64.1			24.4	75.6		
*Ethnicity*				0.8622				0.0872
Non-Caucasian^c^	35.2	64.8	0.96		15.8	84.2	0.50	
Caucasian	36.1	63.9			27.1	72.9		
								
*Sex*				0.5845				0.1300
Female	36.9	63.1	1.13		27.9	72.1	1.63	
Male	34.1	65.9			19.1	80.9		
								
*Marital status*				0.2900				0.8200
No more married	45.5	54.5	1.41		22.9	77.1	0.83	
Never married	33.7	66.3	0.86		22.2	77.8	0.80	
Married	37.1	62.9			26.4	73.6		
								
*Employment status*				0.5970				0.0326
Unemployed and retired	36.4	63.6	0.88		16.0	84.0	0.45	
Employed	39.5	60.5			29.8	70.2		
								
*Education*^d^				0.2155				0.0484
<College	41.1	58.9	1.36		19.3	80.7	0.51	
⩾College	33.9	66.1			32.0	68.0		
								
*Income*^e^				0.2000				0.1100
Low	33.3	66.7	1.02		17.9	82.1	0.48	
Middle	43.0	57.0	1.54		19.8	80.2	0.54	
High	32.8	67.2			31.3	68.8		
								
*Experienced traumatic event*				0.3318				0.5510
No	37.4	62.6	1.25		23.1	76.9	0.83	
Yes	32.5	67.5			26.5	73.5		
								
*Witnessed traumatic event*				0.3520				0.6650
No	36.9	63.1	1.25		23.8	76.2	0.87	
Yes	31.9	68.1			26.5	73.5		
								
*Drug abuse*				0.6800				0.1820
No	35.6	64.4	0.55		19.6	80.4	0.24	
Yes	50.0	50.0			50.0	50.0		
								
*Insurance status*				0.9000				0.1500
No insurance	39.8	60.2	1.12		18.4	81.6	0.49	
Public insurance	40.0	60.0	1.13		0.0	100.0	0.00	
Private insurance	37.2	62.8			31.6	68.4		

	*Mean (s.d.)*	*Mean (s.d.)*	*OR*^b^	P*-value*	*Mean (s.d.)*	*Mean (s.d.)*	*OR*^b^	P*-value*
Age (years; units=10)^f^	39.5 (13.0)	42.4 (13.8)	0.85	0.0565	38.2 (13.4)	42.1 (12.4)	0.78	0.0470
Years of education (units=4)^f^	13.8 (3.4)	14.5 (3.3)	0.78	0.0805	14.6 (3.3)	14.4 (2.9)	1.08	0.7421
Income ($/month; units=1000)^f^	2543 (2445)	2743 (2964)	0.97	0.5208	3506 (4367)	2824 (4682)	1.03	0.3620
Age at onset of first MDE (units=10)^f^	27.0 (15.0)	27.0 (15.2)	1.00	0.9722	25.9 (13.0)	24.9 (14.1)	1.05	0.6320
Number of MDEs (units=5)^f^	3.7 (6.8)	6.1 (8.0)	0.76	0.0152	4.2 (7.5)	6.6 (11.7)	0.86	0.1980
QIDS-SR_16_ (difference W4-Enroll; units=1)^f^	−6.7 (4.2)	−5.9 (3.7)	0.95	0.0765	−6.4 (4.2)	−6.6 (3.8)	1.01	0.7392

Abbreviations: MDE, major depressive episode; OR, odds ratio; QIDS-SR16, Quick Inventory of Depressive Symptomatology Self-Report; REM, remission.

a Remission was defined as a score ⩽5 at week 6 on the 16-item QIDS-SR16.

b For characteristics for which the measurement is categorical, the comparison is with the group listed last under the category or with the opposite characteristic. For characteristics for which the measurement is continuous, the odds ratio is relative to an increase in the measurement by the number of units indicated.

c Asian, American Indian or Alaskan Native, Native Hawaiian/Other Pacific Islander or multiracial.

d <College=⩽16 years of schooling; ⩾College=⩾16 years of schooling.

e Low=monthly gain ⩽$1000; Middle=monthly gain $1000–$2500; High=monthly gain>$2500.

f Units are relevant to odds ratios only OR, MDE.
