# Supplementary material for: Citalopram amplifies the influence of living conditions on mood in depressed patients enrolled in the STAR*D study
Source: Transl Psychiatry. 2017 Mar 21;7(3):e1066–. doi: 10.1038/tp.2017.35 (PMC5416678; doi:10.1038/tp.2017.35)
Supplement: Supplementary Information [file tp201735x1.pdf]

Citalopram amplifies the influence of living conditions on mood in a dose-dependent manner in depressed patients enrolled in the STAR*D study

Flavia Chiarotti, Aurelia Viglione, Alessandro Giuliani, and Igor Branchi

SUPPLEMENTARY DATA

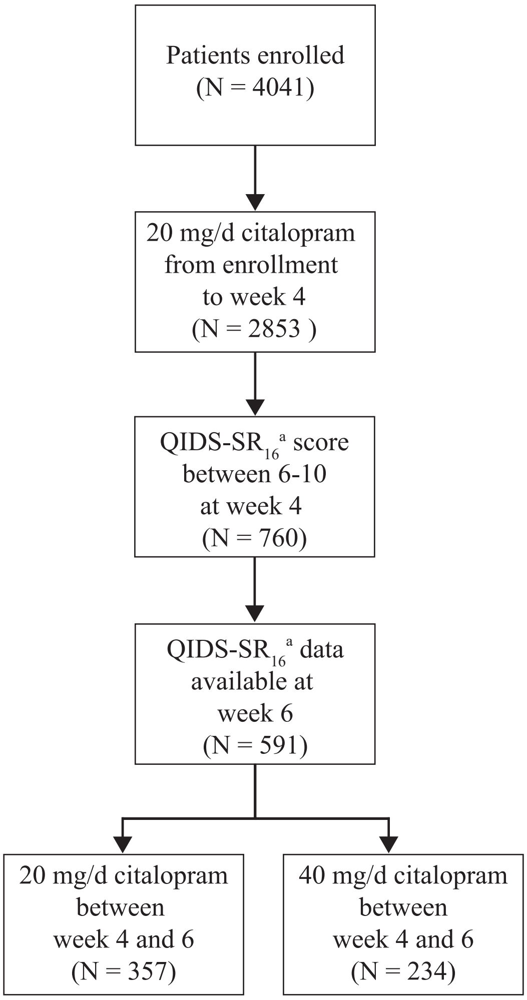

[a] QIDS-SR$_{16}$ = Quick Inventory of Depressive Symptomatology-Self-Report

Figure S1: Criteria used to select patients participating to the STAR*D study which constitute the evaluable sample analyzed in the present study

TABLE S1. QIDS-SR16 values at enrollment and week 4 in the 20 and 40 mg/d dose groups

	20mg/d	40mg/d
**Enrollment**		
Minimum	3	5
1st Quartile	11	12
Median	14	15
Mean	14.14	14.7
3rd Quartile	17	17
Maximum	23	26
Standard deviation	3.78	3.79
**Week 4**		
Minimum	6	6
1st Quartile	7	7
Median	8	8
Mean	7.93	8.16
3rd Quartile	9	9
Maximum	10	10
Standard deviation	1.39	1.44

TABLE S2. Baseline sociodemographic characteristics associated with improvement by citalopram dose[a] - Relative Risk

Characteristics	20 mg/die *N* = 357 (60.4%) IMPROVED n	NOT IMPR n	RR[b]	95%CI Lower	Upper	40 mg/die *N* = 234 (39.6%) IMPROVED n	NOT IMPR n	RR[b]	95%CI Lower	Upper
**Overall**	203	154				128	106			
**Ethnicity**										
Non-Caucasian[c]	59	49	0.94	0.77	1.16	26	31	0.79	0.58	1.08
Caucasian	144	105				102	75			
**Sex**										
Female	130	92	1.08	0.89	1.31	81	59	1.16	0.90	1.48
Male	73	62				47	47			
**Marital status**										
No more married	55	22	1.19	0.98	1.46	26	22	0.91	0.66	1.24
Never married	49	37	0.95	0.76	1.20	32	22	0.99	0.75	1.31
Married	79	53				52	35			
**Employment status**										
Unemployed & Retired	66	44	0.95	0.79	1.14	36	39	**0.74**	**0.56**	**0.97**
Employed	117	68				74	40			
**Education[d]**										
< College	112	68	1.01	0.84	1.21	57	57	**0.71**	**0.56**	**0.89**
≥ College	71	44				53	22			
**Income[e]**										
Lower	57	48	0.94	0.75	1.18	29	38	**0.64**	**0.47**	**0.88**
Middle	68	46	1.03	0.84	1.27	41	40	**0.75**	**0.58**	**0.98**
Higher	74	54				54	26			
**Experienced traumatic event**										
No	118	85	1.07	0.89	1.29	67	63	0.86	0.68	1.09
Yes	82	69				61	41			
**Witnessed traumatic event**										
No	141	100	1.12	0.91	1.38	85	79	0.82	0.65	1.04
Yes	59	54				43	25			
**Drug abuse**										
No	38	35	1.04	0.26	4.24	26	30	0.62	0.33	1.16
Yes	1	1				3	1			
**Insurance status**										
No insurance	64	39	1.01	0.83	1.22	41	35	0.80	0.62	1.03
Public insurance	17	8	1.10	0.82	1.48	2	12	**0.21**	**0.06**	**0.77**
Private insurance	101	63				66	32			

Table S1

[a]Improvement was measured as a reduction ≥ 1 in 16-item Quick Inventory of Depressive Symptomatology-Self-Report (QIDS-SR_{16}) score between week 4 and 6

[b]Comparison is with the group listed last under the category or with the opposite characteristic

[c]Asian, American Indian or Alaskan Native, Native Hawaiian/Other Pacific Islander, or multiracial;[d]< College = <16 years of schooling , ≥ College = ≥16 years of schooling; [e]Lower **=** monthly gain ≤ $1000, Middle = monthly gain $1000-$2500, Higher = monthly gain> $2500

RR, Relative Risk

TABLE S3. Baseline sociodemographic characteristics associated with remission by citalopram dose[a] - Relative Risk

	20 mg/die *N* = 357 (60.4%)			95%CI		40 mg/die *N* = 234 (39.6%)			95%CI	
**Characteristics**	**REM n**	**NO REM n**	**RR[b]**	**Lower**	**Upper**	**REM n**	**NO REM n**	**RR[b]**	**Lower**	**Upper**
**Overall**	203	154				128	106			
**Ethnicity**										
Non-Caucasian[c]	38	70	0.97	0.72	1.32	9	48	0.58	0.31	1.11
Caucasian	90	159				48	129			
**Sex**										
Female	82	140	1.08	0.81	1.45	39	101	1.45	0.89	2.38
Male	46	89				18	76			
**Marital status**										
No more married	35	42	1.22	0.88	1.70	11	37	0.87	0.46	1.62
Never married	29	57	0.91	0.63	1.32	12	42	0.84	0.46	1.55
Married	49	83				23	64			
**Employment status**										
Unemployed & Retired	40	70	0.92	0.68	1.25	12	63	**0.54**	**0.30**	**0.97**
Employed	73	112				34	80			
**Education[d]**										
< College	74	106	1.21	0.89	1.65	22	92	**0.60**	**0.37**	**0.99**
≥ College	39	76				24	51			
**Income[e]**										
Lower	35	70	1.02	0.70	1.47	12	55	0.57	0.31	1.05
Middle	49	65	1.31	0.95	1.81	16	65	0.63	0.37	1.09
Higher	42	86				25	55			
**Experienced traumatic event**										
No	76	127	1.15	0.86	1.54	30	100	0.87	0.56	1.37
Yes	49	102				27	75			
**Witnessed traumatic event**										
No	89	152	1.16	0.84	1.59	39	125	0.90	0.55	1.45
Yes	36	77				18	50			
**Drug abuse**										
No	26	47	0.71	0.17	2.95	11	45	0.39	0.13	1.20
Yes	1	1				2	2			
**Insurance status**										
No insurance	41	62	1.07	0.79	1.46	14	62	0.58	0.33	1.01
Public insurance	10	15	1.08	0.64	1.81	0	14	0.00	n.d.	n.d.
Private insurance	61	103				31	67			

Table S2

[a]Remission was defined as a score ≤ 5 at week 6 on the 16-item Quick Inventory of Depressive Symptomatology-Self-Report

[b]Comparison is with the group listed last under the category or with the opposite characteristic

[c]Asian, American Indian or Alaskan Native, Native Hawaiian/Other Pacific Islander, or multiracial;[d]< College = <16 years of schooling , ≥ College = ≥16 years of schooling; [e]Lower = monthly gain ≤ $1000, Middle = monthly gain $1000-$2500, Higher = monthly gain> $2500

RR, Relative Risk; n.d., not determined

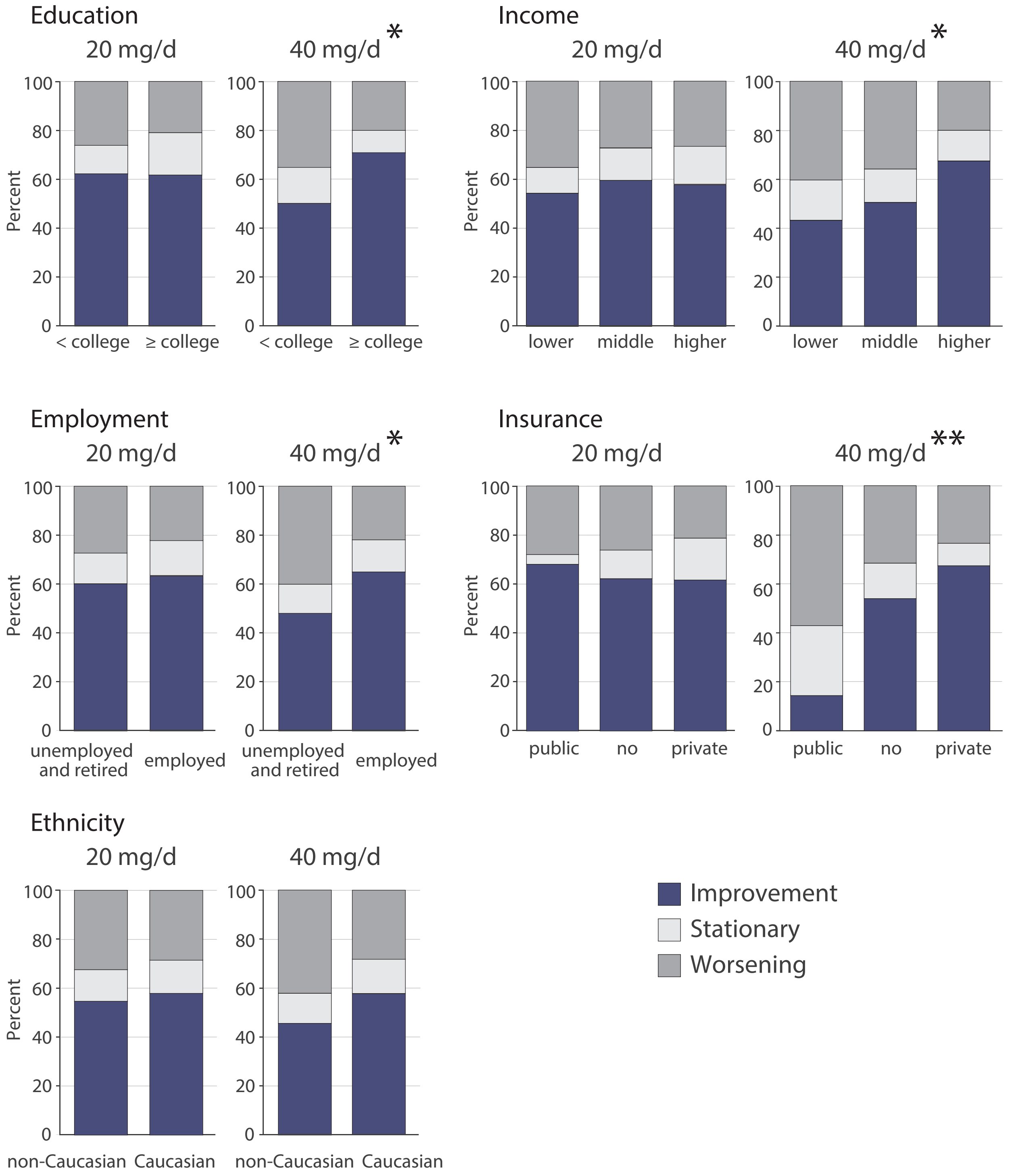

Figure S2. The 40, compared to 20 mg/d dose, leads to an increase not only of the percent of improvements in patients living in a favorable environment but also of and the percent of worsening in patients living in an unfavorable environment. Percent of patients showing improvement (i.e., reduction ≤1 in QIDS-SR16 score), percent of stationary patients (i.e. no change in QIDS-SR16 score) and percent of patients showing a worsening (i.e., increase ≥1 in QIDS-SR16 score) were measured between week 4 and 6, according to the dose regimen - either 20 or 40 mg/d dose. Only patients receiving the 40 mg/d dose showed a variation in these percent significantly moderated by the sociodemographic characteristics. Chi-square *p<0.05 and ** p<0.01
